# High Expression of TMEM33 Predicts Poor Prognosis and Promotes Cell Proliferation in Cervical Cancer

**DOI:** 10.3389/fgene.2022.908807

**Published:** 2022-06-27

**Authors:** Hanxiang Chen, Xia Zhao, Yongqing Li, Shaoming Zhang, Yunshan Wang, Lili Wang, Wanshan Ma

**Affiliations:** ^1^ Department of Clinical Laboratory, The First Affiliated Hospital of Shandong First Medical University and Shandong Provincial Qianfoshan Hospital, Jinan, China; ^2^ Shandong LaiBo Biotechnology Co., Ltd., Jinan, China; ^3^ Department of Clinical Laboratory, Shandong Provincial Qianfoshan Hospital, Cheeloo College of Medicine, Shandong University, Jinan, China; ^4^ Medical Research and Laboratory Diagnostic Center, Jinan Central Hospital, Cheeloo College of Medicine, Shandong University, Jinan, China

**Keywords:** CESC, TMEM33, prognosis, immune infiltration, cell proliferation

## Abstract

**Background:** The prognosis of patients with advanced cervical cancer remains unsatisfactory. A study indicated that transmembrane protein 33 (TMEM33) was implicated in tumor recurrence, while its role in cervical cancer has not been elucidated.

**Methods:** TMEM33 expression in cervical squamous cell carcinoma and endocervical adenocarcinoma (CESC) was primarily screened in The Cancer Genome Atlas (TCGA), and further validated in Gene Expression Omnibus (GEO) database. The Kaplan–Meier plotter analysis and Cox regression were constructed to evaluate the prognostic value of TMEM33 in CESC. Functional enrichment analysis was performed with GO, KEGG and GSEA tools. CCK-8 assay and colony formation assay were performed to investigate the carcinogenesis role of TMEM33 in cervical cancer cell proliferation.

**Results:** TMEM33 expression was significantly elevated in CESC compared with normal tissues. High expression of TMEM33 was associated with poor prognostic clinical characteristics in CESC patients. KM-plotter analysis revealed that patients with increased TMEM33 had shorter overall survival (OS), progress free interval (PFI), and disease specific survival (DSS). Moreover, Multivariate Cox analysis confirmed that high TMEM33 expression was an independent risk factor for OS in patients with CESC. TMEM33 was associated with immune infiltrates, and its expression was correlated with tumorigenesis-related genes RNF4, OCIAD1, TMED5, DHX15, MED28 and LETM1. More importantly, knockdown of TMEM33 in cervical cancer cells decreased the expression of those genes and inhibited cell proliferation.

**Conclusion:** Increased TMEM33 in cervical cancer can serve as an independent prognostic marker and might play a role in tumorigenesis by promoting cell proliferation.

## Introduction

Cervical cancer is the fourth most common cancer among women, with approximately 600,000 new cases and 300,000 deaths reported every year (Sindian et al., 2020). Although the incidence and mortality rates are decreasing due to early diagnosis and vaccines, these methods do not affect regression of advanced cervical cancer. More than 80% of cervical cancer is the result of human papillomavirus (HPV) infection, of which 70% are positive for high-risk HPV type 16 or 18 (Burd, 2003). Persistent infection with HPV induces high grade cervical intraepithelial neoplasia (CIN), which may progress to invasive cervical cancer if left untreated (Koshiol et al., 2008). The International Federation of Gynecology and Obstetrics (FIGO) system is commonly used for cervical cancer staging (Matsuo et al., 2019). However, significant differences in survival are observed in the same FIGO staging, suggesting that more prognostic markers are needed for reflecting cancer biodiversity, improving individual risk stratification, and for directing a personalized treatment regimen.

Transmembrane protein 33 (TMEM33) is a three-pass transmembrane domain protein conserved throughout evolution (Chadrin et al., 2010). Human TMEM33 has been shown to locate in the nuclear envelope and endoplasmic reticulum (ER) *in vitro*, serve as a regulator of the tubular ER network by suppressing the membrane-shaping activity of reticulons (Sakabe et al., 2015). At present, limited functional studies of TMEM33 in multicellular organisms have been reported. Savage AM. et al. characterized TMEM33 is required in an EC-specific manner for Vegfa-mediated Ca2+ oscillations, to promote angiogenesis in zebrafish embryos (Savage et al., 2019). TMEM33 also regulates intracellular calcium homeostasis in a polycystin-2 (PC2)-dependent manner, causes cathepsins translocation and sensitization to apoptosis in renal tubular epithelial cells (Arhatte et al., 2019). Recently, TMEM33 was shown as a downstream effector of PKM2 in regulating the activation of SREBPs and lipid metabolism (Liu et al., 2021). Although the role of TMEM33 is gradually revealed in different aspects, its expression in cancer and implications in tumorigenesis are largely unknown, warranting further exploration.

In the present study, we identified TMEM33 was markedly overexpressed in Cervical Squamous Cell Carcinoma and Endocervical Adenocarcinoma (CESC) from The Cancer Genome Atlas (TCGA) and Gene Expression Omnibus (GEO) dataset. The aberrant expression was verified by Immunoblotting and RT-qPCR in cervical cancer cells. The common biological pathways and enrichment analysis of the differentially expressed genes were performed by GO, KEGG and GSEA. The interacted protein network and correlated genes were explored utilizing the STRING and the GEPIA2 websites, respectively. Subsequently, the prognostic value of TMEM33 in cervical cancer and its role in immune infiltration and cell proliferation were evaluated.

## Materials and Methods

### TCGA, GTEx and GEO Dataset

Clinical information of CESC patients and gene expression quantification data were downloaded from the TCGA dataset. Gene expression data with clinical information on healthy cervix tissue were obtained from the Genotype-Tissue Expression (GTEx) database. The gene expression level was qualified by UCSC Xena Toil RNAseq pipeline (Vivian et al., 2017). A total of 306 cervical cancer samples and 13 healthy cervix controls were enrolled in the present study. GSE63514 (with 24 normal cervix tissues and 28 cervical cancer tissues) was used as a validation dataset (Yi et al., 2020). Gene expression and clinical data of patients in GSE63514 were downloaded from the GEO database. No extra Ethics committee approval was necessary since the data were downloaded from public databases.

### Data Processing

R programming language (version 3.6.3) was the principal tool for analyzing data throughout the study. The differentially expressed genes between cervical cancer samples and healthy controls with different clinical stages were identified using DESeq2 package (version1.26.0) (Love et al., 2014). GO and KEGG analysis were conducted by the clusterProfiler package (version 3.14.3), and visualized by the ggplot2 package. GSEA analysis was performed using the clusterProfiler package (version 3.14.3) (Yu et al., 2012). Survival analysis and survival curves were done by the survminer packages (version 0.4.9). Survival pROC package (version 1.17.0.1) was used to create a ROC curve. Nomogram and calibration analysis were generated using rms package (version 6.2-0) (Liu et al., 2018). Immune infiltration analysis was performed by the GSVA package (version 1.34.0) (Hänzelmann et al., 2013).

### STRING, GEPIA2 and HPA Websites

STRING website^1^ hosts a large collection of integrated and consolidated protein-protein interaction data. We imported TMEM33 with the main settings such as meaning of network edges “evidence”, active interaction sources “experiments”, the minimum required interaction score “Low confidence (0.150)”, and max number of interactors to show “no more than 50 interactors” operated.

GEPIA2 website^2^ is a web server supporting normal and cancer gene expression profiling and interactive analysis. The top 200 TMEM33 correlated genes in CESC were obtained via “similar gene detection” module.

The Human Protein Atlas (HPA)^3^ is a website contains immunonhistochemistry-based expression data for approximately 20 highly common types of cancers. The protein expression of TMEM33 between CESC tissue and normal cervix tissue was shown by immunohistochemistry image.

### Cell Culture, RNAi and Cell Transfection

Cervical cancer cell lines HeLa, SiHa, CaSki, C33A and normal cervix-derived cell line H8 were purchased from ATCC. All cell lines were cultured in a complete medium with 10% FBS (Gibco, United States) as previously described (Cui et al., 2020). For cell transfection, cells were seeded onto a 60 mm dish and transfected with a final concentration of 20 nM siRNA per target gene using Lipofectamine 3,000 transfection reagents according to the manufacturer’s instruction (Invitrogen, United States). The siRNA sequences (Ruibo, China) were shown in Supplementary Table S1.

### Real-Time Quantitative PCR and Immunoblotting

RT-qPCR and immunoblotting were performed as described previously (Chen et al., 2017). All the primers were synthesized by Biosune, with the primer sequences listed in Supplementary Table S1. The TMEM33 polyclonal antibody (Bethyl, United States) was used at 1:1000.

### CCK-8, Colony Formation Assay and EDU Incorporation Assay

The cell viability was determined by the Cell Counting Kit-8 (Apexbio, United States) according to the manufacturer’s instruction and read by the Multiskan GO microplate reader (ThermoFisher, United States ) at 450 nm. An amount of 800 cells per well was seeded in six-well plates and incubated for 14 days until colonies appeared. The colonies were fixed with methanol and stained with 0.5% crystal violet solution.

DNA synthesis was analyzed by using the EDU cell proliferation image kit (Abbkine, China). Briefly, 1 × 10^4^ cervical cancer cells were cultured in 96-well plates in triplicate after transfection. cells were incubated with 50 μmol EdU for 2 h at 37°C, fixed with 4% paraformaldehyde for 30 min, then treated with 0.5% Triton X-100 for 10 min. The proliferation rate was determined under a fluorescence microscope.

### Statistical Analysis

R programming language and GraphPad prism 7.0 software were used for statistical analysis. Student’s t-test was used to compare differences between two groups. Univariate Cox regression and multivariate Cox regression analysis were performed to evaluate the prognostic value of TMEM33 with CESC. Kaplan-Meier curve was used to assess differences in overall survival, disease specific survival and progress free interval between high-risk score group and low-risk score group, the significance was determined by log-rank test. Hazard ratios (HR) and 95% confidence intervals (CI) were calculated to evaluate variables associated with overall survival. *p* < 0.05 was used as a cutoff value for significance.

## Results

### TMEM33 Is Highly Expressed in Cervical Squamous Cell Carcinoma and Endocervical Adenocarcinoma

**FIGURE 1:**
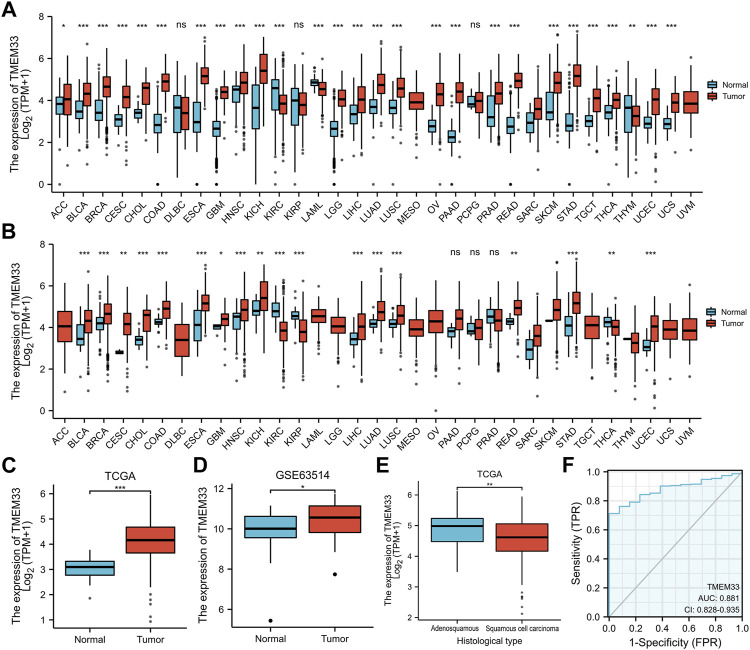
TMEM33 expression in pan-cancers and CESC. Box plots assessing the expression profiles of TMEM33 between tumor and normal tissue **(A)** or between tumor and pericarcinous tissue **(B)** in different types of cancers based on TCGA and GTEx datasets. **(C)** The expression of TMEM33 in normal cervical tissue and CESC tissue. **(D)** The validation of TMEM33 expression in GSE63514 dataset. **(E)** The expression profile of TMEM33 based on histological type of CESC. **(F)** Receiver operating characteristic (ROC) curve analysis evaluating the performance of TMEM33 for CESC diagnosis. *p < 0.05, **p < 0.01, and ***p < 0.001.

Based on the TCGA and GTEx databases, we examined the expression profile of TMEM33 in pan-cancer analysis. As shown in Figure 1A, among 33 cancer types, TMEM33 was significantly highly expressed in 24 cancers compared with normal tissues. For TCGA tumors and cancer-adjacent normal tissues, TMEM33 expression was significantly up-regulated in 14 cancer types (Figure 1B). Specifically, TMEM33 expression was increased in CESC (Figure 1C), and was further validated in the GSE63514 dataset (Figure 1D). The expression of TMEM33 was higher in adenosquamous compared with squamous cell carcinoma of CESC (Figure 1E). In order to explore the clinical value of TMEM33 in CESC diagnosis, we performed ROC curve analysis. As the area under the curve (AUC) was 0.881, TMEM33 showed significantly high sensitivity and specificity for CESC diagnosis (Figure 1F).

### Association of TMEM33 Expression and Clinicopathological Characteristics in CESC

We examined the clinicopathological characteristics of CESC patients based on the mean value of TMEM33 expression. As shown in Table 1, the high expression of TMEM33 was significantly associated with Height (*p* = 0.048), N stage (*p* = 0.003) and Histological type (*p* = 0.003), while it was not associated with other features. The results of univariate analysis using logistic regression indicated that TMEM33 expression was correlated with poor prognostic clinical characteristics in CESC patients (Table 2). High TMEM33 expression was correlated with N stage (OR = 0.372, *p* = 0.002) and Histological type (OR = 0.366, *p* = 0.002).

**TABLE 1:** Clinicopathological characteristics of CESC patients with differential TMEM33 expression.

Characteristic	Levels	Low Expression of TMEM33	High Expression of TMEM33	*p* Value
n		153	153	
Age, n (%)	≤50	99 (32.4%)	89 (29.1%)	0.291
	>50	54 (17.6%)	64 (20.9%)	
Weight, n (%)	≤70	61 (22%)	77 (27.8%)	0.063
	>70	78 (28.2%)	61 (22%)	
Height, n (%)	≤160	59 (22.5%)	75 (28.6%)	0.048*
	>160	73 (27.9%)	55 (21%)	
T stage, n (%)	T1	71 (29.2%)	69 (28.4%)	0.121
	T2	36 (14.8%)	36 (14.8%)	
	T3	14 (5.8%)	7 (2.9%)	
	T4	2 (0.8%)	8 (3.3%)	
N stage, n (%)	N0	58 (29.7%)	76 (39%)	0.003**
	N1	41 (21%)	20 (10.3%)	
M stage, n (%)	M0	53 (41.7%)	63 (49.6%)	0.410
	M1	7 (5.5%)	4 (3.1%)	
Clinical stage, n (%)	Stage I	77 (25.8%)	85 (28.4%)	0.234
	Stage II	34 (11.4%)	35 (11.7%)	
	Stage III	29 (9.7%)	17 (5.7%)	
	Stage IV	9 (3%)	13 (4.3%)	
Histological type, n (%)	Adenosquamous	16 (5.2%)	37 (12.1%)	0.003**
	Squamous cell carcinoma	137 (44.8%)	116 (37.9%)	
Age, median (IQR)		45 (38, 56)	47 (39, 56)	0.221

*p < 0.05, **p < 0.01.

**TABLE 2:** Logistic regression analysis of association between clinicopathological characteristics and TMEM33 expression in CESC patients.

Characteristics	Total(N)	Odds ratio (OR)	*p* Value
T stage (T2&T3&T4 vs. T1)	243	1.009 (0.606-1.680)	0.972
N stage (N1 vs. N0)	195	0.372 (0.194-0.695)	0.002**
M stage (M1 vs. M0)	127	0.481 (0.120-1.681)	0.263
Clinical stage (Stage II&Stage III&Stage IV vs. Stage I)	299	0.818 (0.518-1.289)	0.387
Histological type (Squamous cell carcinoma vs. Adenosquamous)	306	0.366 (0.189-0.681)	0.002**

**p < 0.01.

### Identification of TMEM33 as an Independent Prognostic Indicator in CESC

**FIGURE 2:**
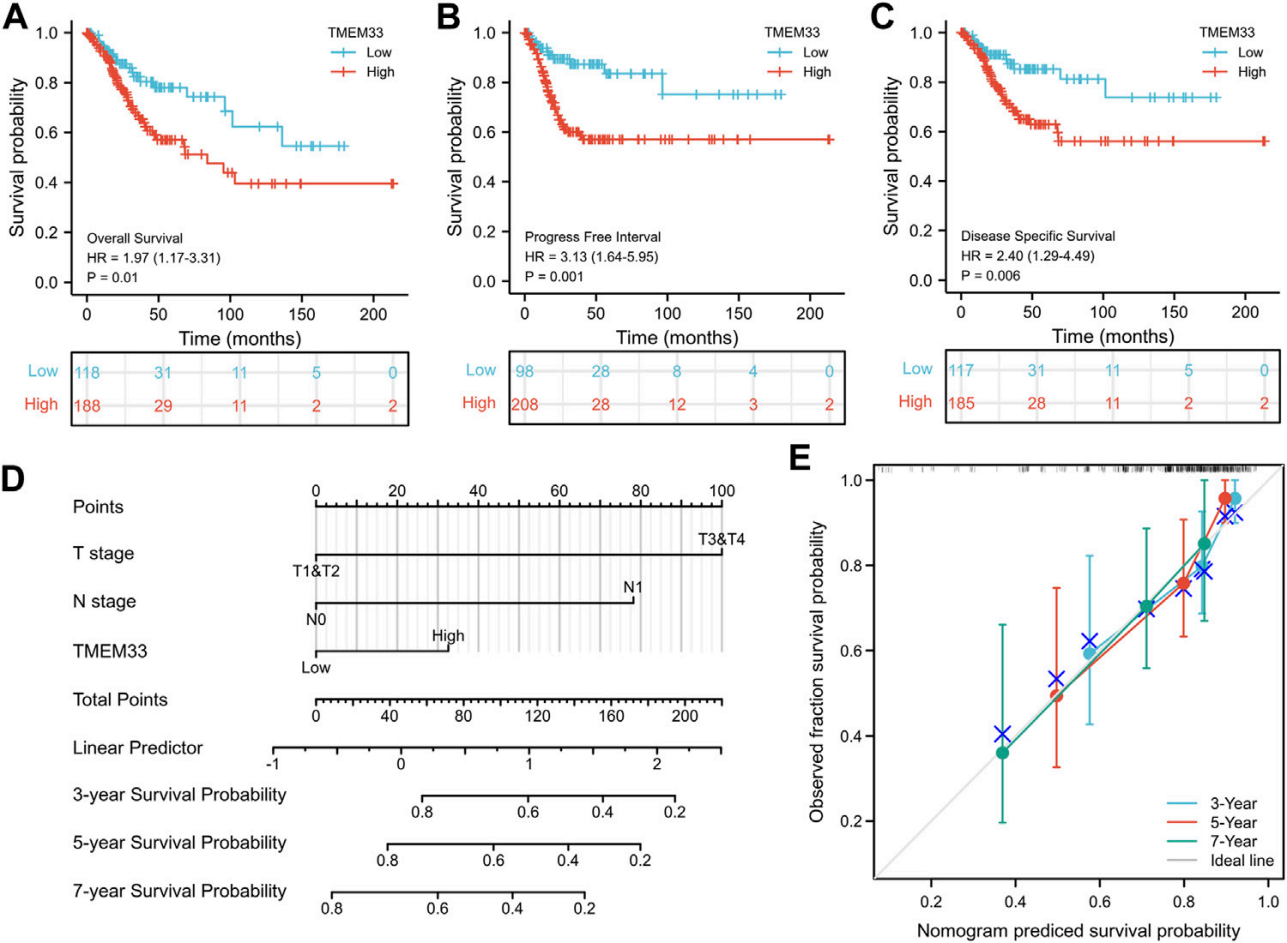
Prognostic value of TMEM33 in CESC based on the TCGA database. Kaplan-Meier analysis comparing overall survival **(A)**, progression-free interval **(B)**, and disease-specific survival **(C)** between low- and high-TMEM33 expression groups. **(D)** A nomogram for predicting probability of patients with 3-, five- and 7-years overall survival. **(E)** Calibration plots validating the efficiency of nomograms for overall survival.

Next, Kaplan-Meier analysis was used to assess the predictive value of TMEM33 on clinical outcomes. As shown in Figures 2A–C, high expression of TMEM33 predicted poor prognosis in both overall survival (OS, HR: 1.97, *p* = 0.01), progress free interval (PFI, HR: 3.13, *p* = 0.001) and disease specific survival (DSS, HR: 2.40, *p* = 0.006). In addition, we conducted univariate and multivariate analysis using the Cox proportional hazards regression model. As shown in Table 3 and Supplementary Figure S1, the univariate cox regression analysis revealed TMEM33, clinical T, N and M stages were positive prognostic factors informing worsen CESC patient overall survival. However, only T stage, N stage and TMEM33 were independent risk factors for overall survival in multivariate regression cox analysis.

**TABLE 3:** Cox regression analysis for clinical outcomes in CESC patient.

Characteristics	Total(N)	Univariate Analysis		Multivariate Analysis	
		Hazard Ratio (95% CI)	*p* Value	Hazard Ratio (95% CI)	*p* Value
Age	306				
≤50	188	References			
>50	118	1.289 (0.810-2.050)	0.284		
Weight	277				
≤70	138	References			
>70	139	0.761 (0.463-1.248)	0.279		
Height	262				
≤160	134	References			
>160	128	1.094 (0.635-1.888)	0.746		
T stage	243				
T1	140	References			
T2	72	1.152 (0.562-2.359)	0.700	0.000 (0.000-Inf)	0.992
T3	21	2.710 (1.167-6.292)	0.020*	0.904 (0.117-6.994)	0.923
T4	10	8.088 (3.419-19.135)	<0.001***	84.580 (7.056-1013.898)	<0.001***
N stage	195				
N0	134	References			
N1	61	2.844 (1.446-5.593)	0.002**	2.760 (1.023-7.442)	0.045*
M stage	127				
M0	116	References			
M1	11	3.555 (1.187-10.641)	0.023*	0.925 (0.000-Inf)	1.000
TMEM33	306				
Low	153	References			
High	153	1.692 (1.053-2.718)	0.030*	3.739 (1.189-11.758)	0.024*

CI, confidence interval.

*p < 0.05, **p < 0.01, and ***p < 0.001.

Moreover, a nomogram was constructed taking into account the statistically significant prognostic factors in multivariate Cox regression analysis to predict 3, five and 7-years overall survival (Figure 2D). The calibration plots demonstrated good agreement between prediction and observed events (Figure 2E). These data illustrate that the high expression of TMEM33 predicts poor prognosis of CESC.

### Functional Enrichment Analysis of Differentially Expressed Genes of TMEM33

To better understand the potential function and molecular pathways of the TMEM33 gene in CESC, we analyzed the differentially expressed genes (DEGs) based on the TCGA dataset. A total of 1,178 genes related to TMEM33 expression were altered (711 up-regulated, 467 down-regulated, |log2(FC)|>1 & p. adj<0.05), which may reflect the potential value of TMEM33 on CESC pathogenesis (Volcano plot of the DEGs was shown in Supplementary Figure S2. The DEGs list was shown in Supplementary Table S2).

**FIGURE 3:**
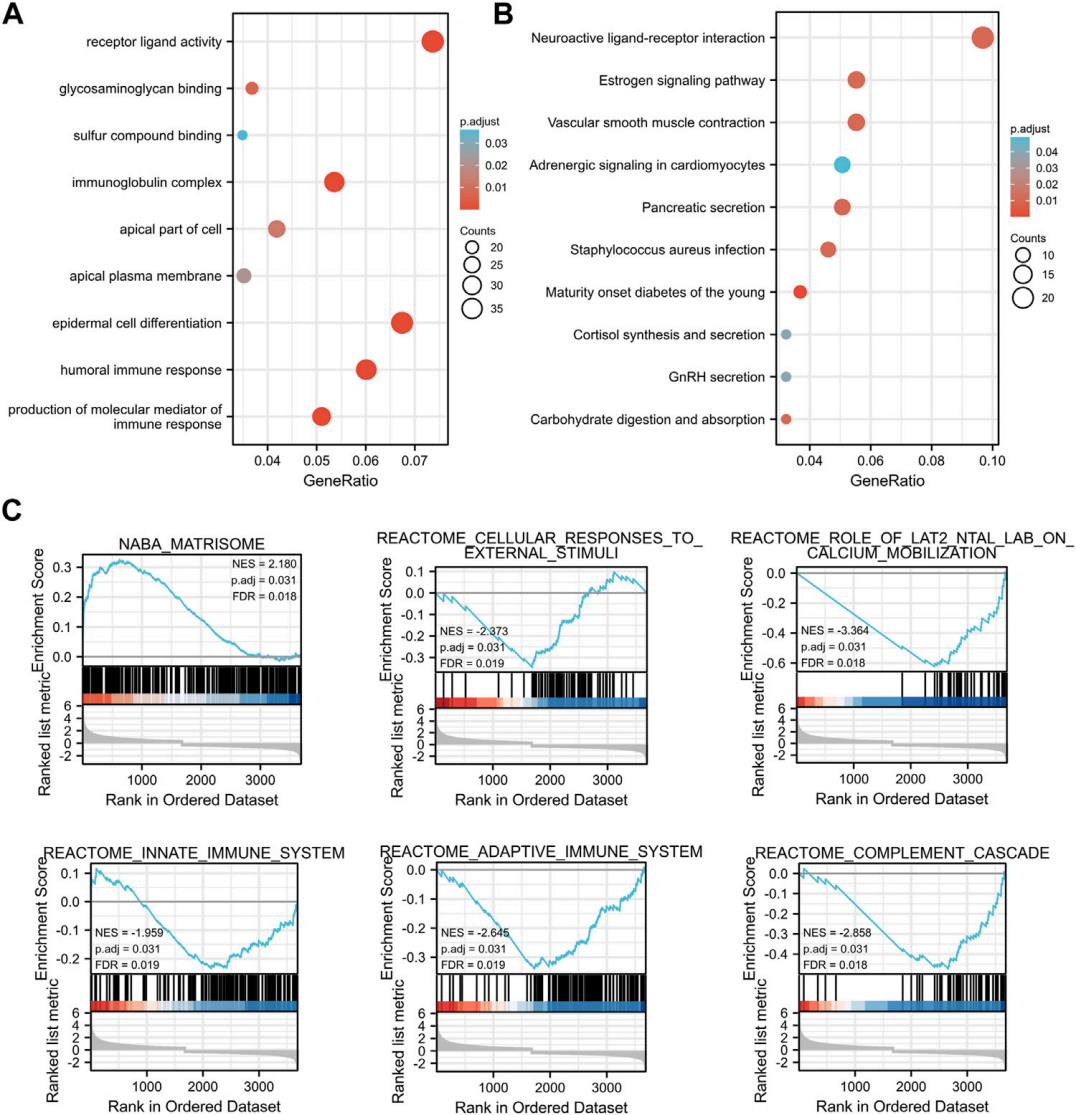
Enrichment analysis of TMEM33-correlated DEGs in CESC. Bubble plots display the functional enrichment results by TMEM33 expression correlated DEGs in terms of biological processes, molecular functions, cellular components **(A)**, and KEGG signaling pathways **(B)**. The functional categories and pathways were annotated with color gradient bubbles of different sizes. **(C)** Significantly enriched pathways with p. adj<0.05, such as matrisome, cellular responses to external stimuli, role of LAT2/NTAL/LAB on calcium mobilization, innate immune system, adaptive immune system, and complement cascade.

Subsequently, TMEM33 and its DEGs were subjected to Gene Ontology (GO) and Kyoto Encyclopedia of Genes and Genomes (KEGG) functional enrichment analysis. As shown in Figure 3A, the gene clusters were involved in epidermal cell differentiation, production of the molecular mediator of immune response, and immunoglobulin production in the category of biological processes (BP). For molecular function (MF), the DEGs were enriched at receptor ligand activity, glycosaminoglycan binding and immunoglobulin receptor binding, etc. In the category of cellular components (CC), the DEGs were mostly distributed in immunoglobulin complex, apical part of cell and apical plasma membrane. The KEGG pathway analysis showed that the DEGs were enriched in neuroactive ligand-receptor interaction, estrogen signaling pathway, vascular smooth muscle contraction, etc. (Figure 3B). The GO and KEGG analysis demonstrate that TMEM33 might participate in signal transduction pathways and immune regulation.

Using the low- and high-TMEM33 expression datasets, we then performed GESA to identify the most significantly altered pathways based on the gene sets from the MSigDB (c2. cp.kegg.v6.2. symbols.gmt). Interestingly, in addition to the pathways that have been reported to reflect the biological functions of TMEM33 as a transmembrane protein, some immune-related signaling pathways were enriched, such as innate immune system, adaptive immune system and complement cascade, with the threshold of NES >1.5 and p. adj<0.05 (Figure 3C), which provided additional evidence that TMEM33 might be implicated in immune response.

### Association of TMEM33 and Immune Cell Infiltration in CESC

**FIGURE 4:**
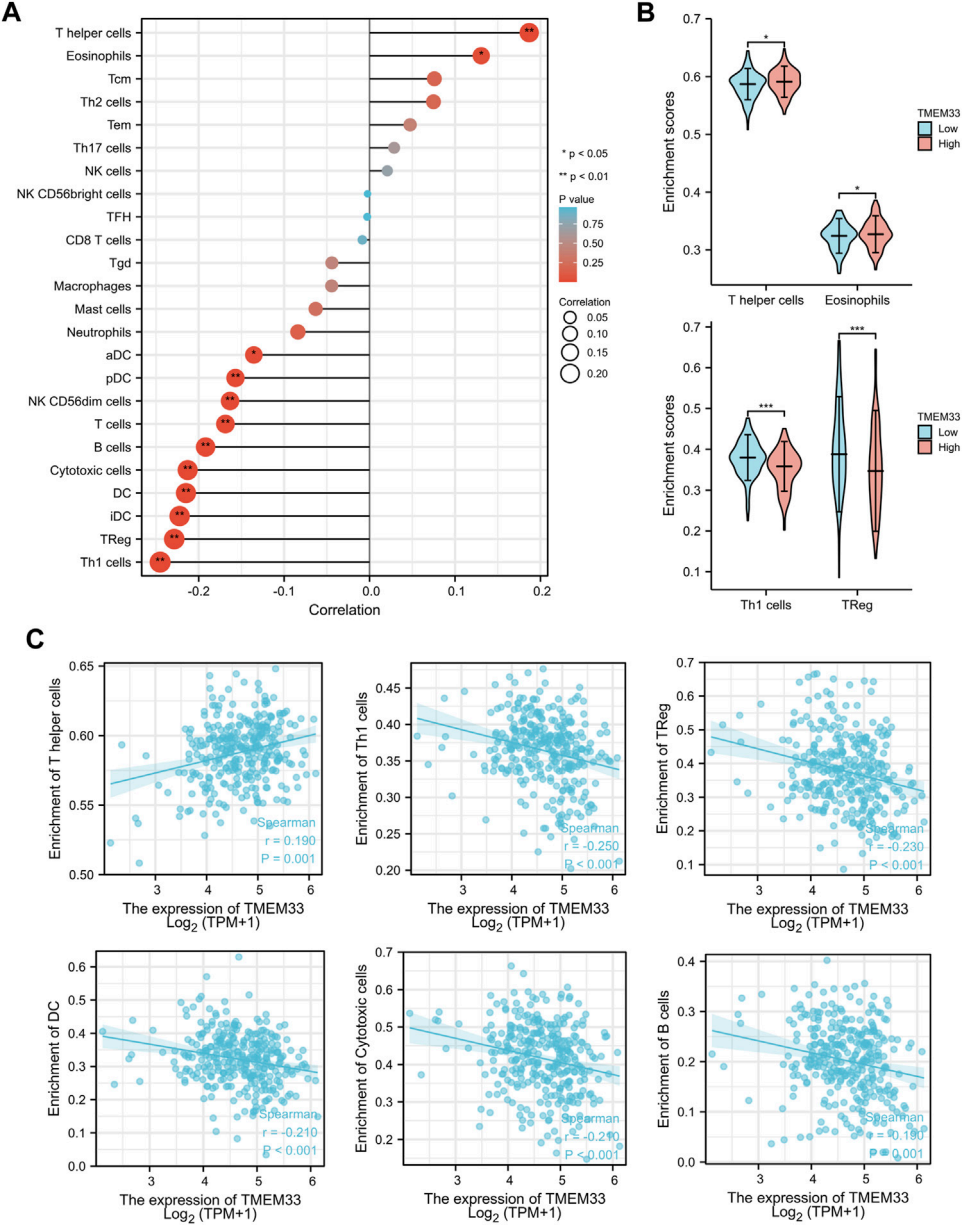
Immune infiltration analysis of TMEM33 in CESCC. **(A)** The forest plot shows the correlation between TMEM33 expression level and 24 immune cells. **(B)** The enrichment scores of TMEM33 expression in T helper cells, Eosinophils, Th1 cells, and TReg. **(C)** The correlation between TMEM33 expression and T helper cells, Th1 cells, TReg, DC, Cytotoxic cells, and B cells. *p < 0.05, **p < 0.01, and ***p < 0.001.

To confirm our hypothesis, we determined the infiltration of 24 immune cell types in CESC using the ssGSEA method, and subsequently the association between TMEM33 and immune cell infiltration was investigated by Spearman’s correlation analysis. As shown in Figure 4A, T helper cells and Eosinophils were positively correlated with TMEM33 expression. However, Th1 cells, Treg, iDC, DC, Cytotoxic cells, B cells, T cells, CD56dim NK cells, pDC and aDC showed a significant negative association with TMEM33. More specifically, we assessed the infiltration levels of the top two relevant immune cells of each group, T helper cells, Eosinophils, Th1 cells and Treg in distinct TICRR groups, which showed consistent trends with the forest plots (Figure 4B). Further, the correlation between TMEM33 expression and T helper cells, Th1 cells, Treg, DC, Cytotoxic cells and B cells in CESC were exhibited by scatter plots (Figure 4C).

### Functional Inference of TMEM33 in CESC

**FIGURE 5:**
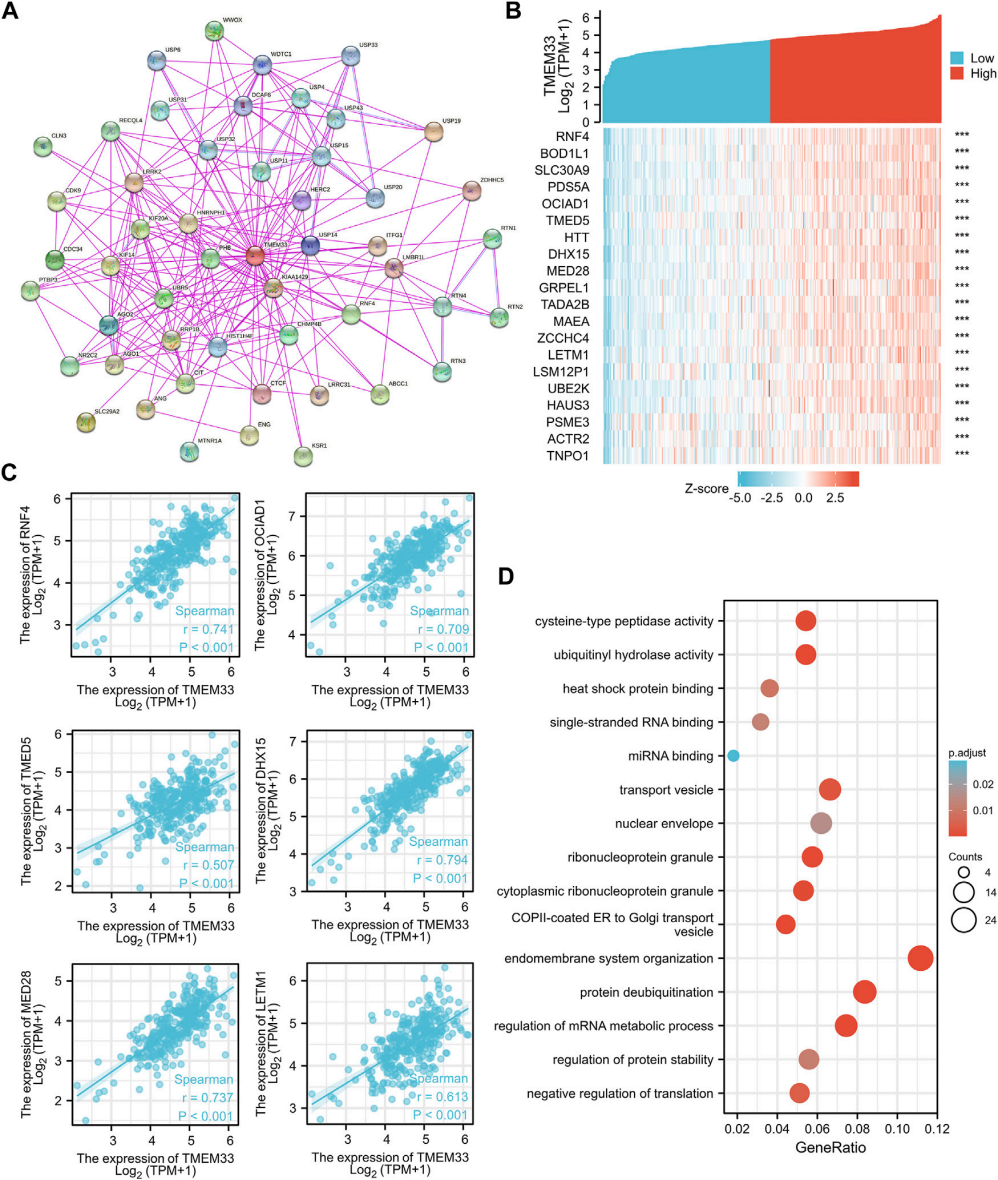
Analysis of TMEM33 correlated genes and PPI network. **(A)** The visualizing interaction network of TMEM33-binding proteins was generated base on STRING database. The purple line indicates “experimentally determined”, the light blue line refers to “protein homology”. **(B)** Top 20 genes positively related to TMEM33 obtained from GEPIA2 were displayed in heatmap. **(C)** Correlation analysis between TMEM33 expression and screened tumorigenesis-related genes, including RNF4, OCIAD1, TMED5, DHX15, MED28, and LETM1. **(D)** GO enrichment analysis by TMEM33-similar and interacted genes. ***p < 0.001.

In order to investigate the internal mechanism of TMEM33 in tumorigenesis, the PPI network analysis was performed by utilizing the STRING database. As shown in Figure 5A, the interaction network of 50 TMEM33-binding proteins with the experimental evidence identification was visualized. In addition, we analyzed the similar genes of TMEM33 in CESC using the “similar gene detection” module of GEPIA2. Corresponding hierarchical clustering analysis of the top 20 similar genes was displayed by the heatmap (Figure 5B). Through comparing the 50 TMEM33-interacted genes and the 200 TMEM33-similar genes, we screened out the common molecule RNF4 (Supplementary Figure S3), which plays a role in protein ubiquitination (Tatham et al., 2008). Moreover, the TMEM33 expression level was remarkably positively correlated with similar genes of RNF4, OCIAD1, TMED5, DHX15, MED28 and LETM1 (Figure 5C), which have been reported to be implicated in tumorigenesis of various cancers (Sengupta et al., 2008; Thomas et al., 2016; Hsieh et al., 2019; Xie et al., 2019; Ji and Hu, 2020; Zou et al., 2021).

**FIGURE 6:**
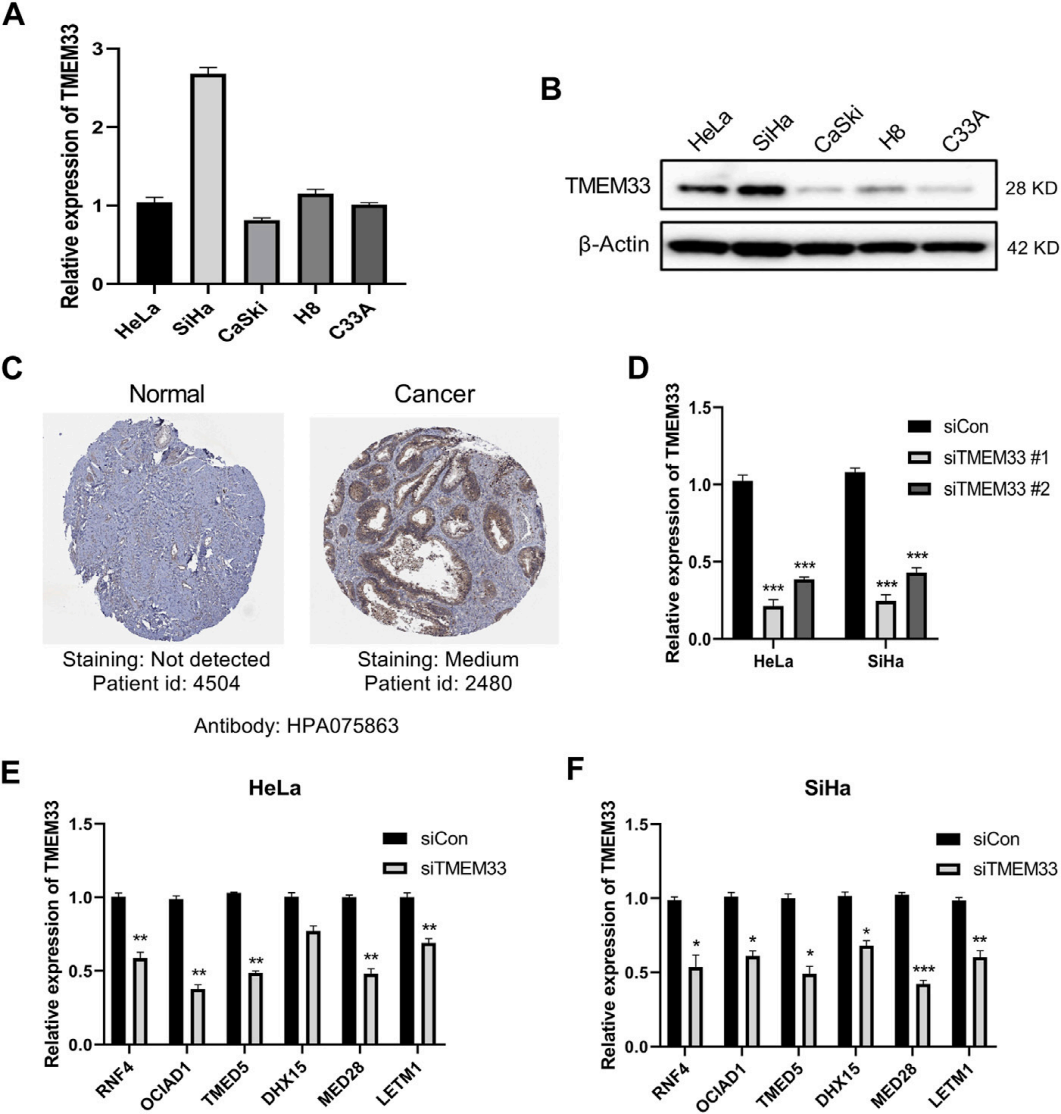
TMEM33 correlates with tumorigenesis-related genes in cervical cancer cells. **(A)** RT-qPCR analysis of relative mRNA level of TMEM33 in cervical cancer cell lines (HeLa, SiHa, CaSki, H8 and C33A). **(B)** Immunoblotting analysis of TMEM33 expression in cervical cancer cell lines. **(C)** Representative immunohistochemistry images of TMEM33 in CESC and normal cervix tissues derived from HPA database. **(D)** siTMEM33 (20 nM) and siCon (20 nM) were transfected with Lipofectamine 2000 according to the manufacturer’s protocol. At 48 h post-transfection, the efficiency was determined by RT-qPCR. **(E,F)** RT-qPCR analysis of relative mRNA level of RNF4, OCIAD1, TMED5, DHX15, MED28 and LETM1 in HeLa and SiHa cells transfected with TMEM33 siRNA. Data are mean ± SD of three independent experiments, *p < 0.05, **p < 0.01, and ***p < 0.001.

We further performed enrichment analysis using the above 250 TMEM33-interacted and similar genes. The top five enrichment pathways of MF, CC and BP were shown in Figure 6E. However, there was no pathway enriched with KEGG owing to the few number of genes selected.

### TMEM33 Expression Is Correlated With the Screened Tumorigenesis-Related Genes in Cervical Cancer Cells

To investigate the expression level of TMEM33 in cervical cancer cells, we performed RT-qPCR and immunoblotting in cervical cancer cell lines HeLa, SiHa, CaSki, H8 and C33A. TMEM33 was up-regulated in SiHa at the mRNA level (Figure 6A), while highly expressed in HeLa and SiHa at the protein level (Figure 6B). Based on that analysis, HeLa (HPV 18+) and SiHa (HPV 16+) cells were chosen for further functional studies. We also examined the protein expression patterns of TMEM33 by the Human Protein Atlas. As shown in Figure 6C, TMEM33 was not detected in normal cervix tissue while medium expressed in cervical cancer tissue.

Since six tumorigenesis related TMEM33-correlated genes were filtered by bioinformatics tools (Figure 5D), we verified their association with TMEM33 in cervical cancer cells. TMEM33 knockdown was performed via RNAi and the transfection efficiency was shown in Figure 6D. As shown in Figures 6E, F, the mRNA expression of RNF4, OCIAD1, TMED5, MED28 and LETM1 were significantly decreased in HeLa-siTMEM33 cells compared with control cells, while all six genes were significantly down-regulated in SiHa-siTMEM33 cells, indicating a certain correlation existed.

### TMEM33 Promotes Cell Proliferation of Cervical Cancer Cells *in vitro*

**FIGURE 7:**
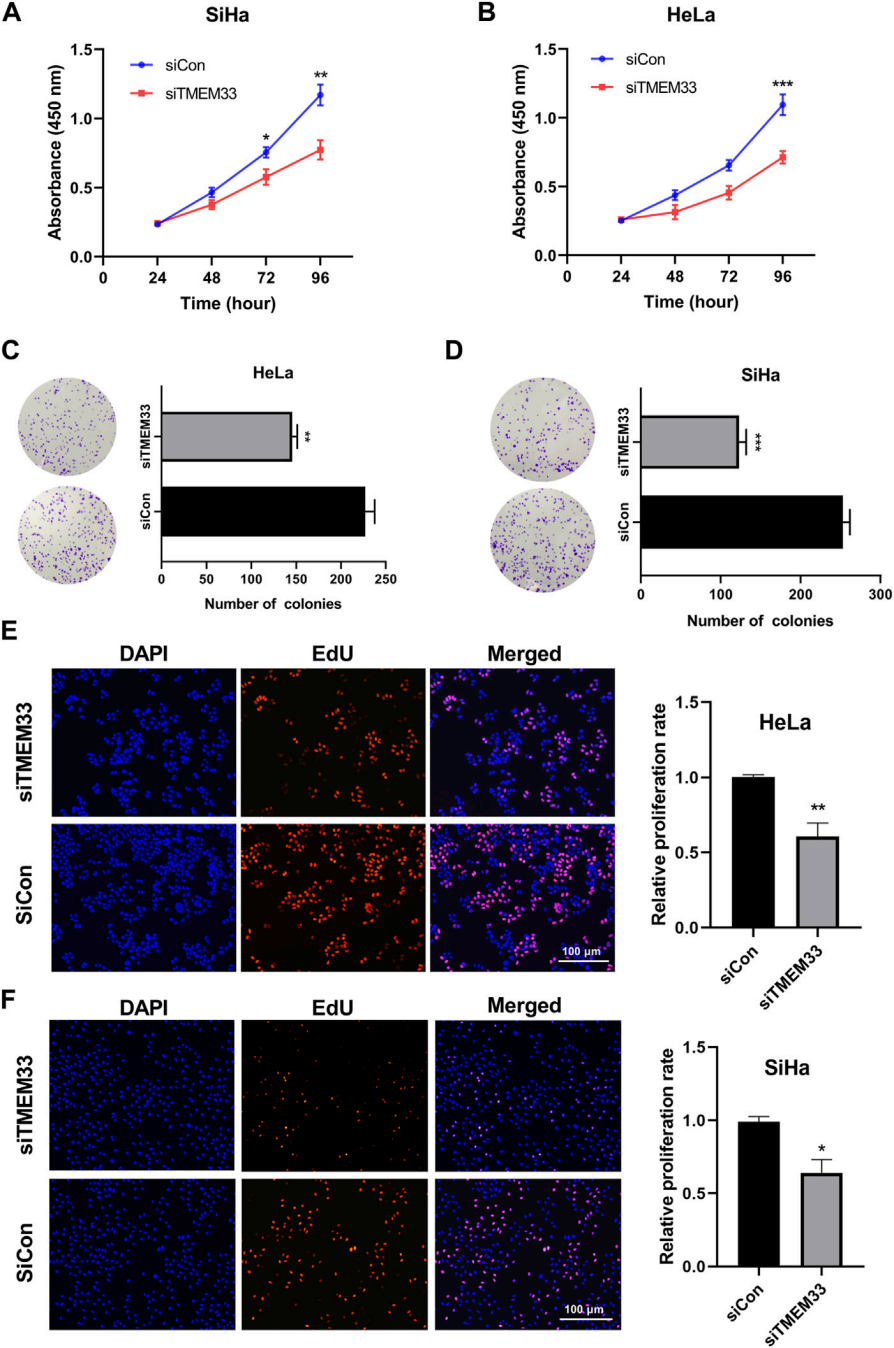
TMEM33 promotes cell proliferation in cervical cancer. **(A,B)** CCK-8 assay of HeLa and SiHa cells transfected with TMEM33 siRNA. **(C,D)** Colony formation assay of HeLa and SiHa cells transfected with TMEM33 siRNA. **(E,F)** DNA synthesis determined by EdU incorporation assay after cell transfection with TMEM33 siRNA in HeLa and SiHa cells (EdU% represents the proportion of EdU-positive cells [orange]). Data are mean ± SD of three independent experiments *p < 0.05, **p < 0.01, and ***p < 0.001.

To further investigate the oncogenic role of TMEM33, we examined the cell viability using CCK-8 assay. HeLa-siTMEM33 and SiHa-siTMEM33 cells showed a significant reduction of cell viability compared with control cells after siRNA transfection in 96 h (Figures 7A, B). In addition, colony formation assay showed that TMEM33 knockdown reduced the clonogenicity of HeLa cells (Figure 7C) and SiHa cells (Figure 7D).

Next, we performed the EDU incorporation assay to demonstrate the effect of TMEM33 on cell proliferation. Knockdown of TMEM33 significantly decreased the relative proliferation rate in both HeLa and SiHa cells (Figures 7E, F). These results suggest that TMEM33 contributes to cell proliferation in cervical cancer, which provide evidence of the carcinogenesis role of TMEM33 in CESC.

## Discussion

The incidence and mortality rates of cervical cancer are still high in developing or least-developed economies. The strategy of cancer treatment depends on pretreatment disease status according to FIGO stage. Despite the early stage of cervical cancer can be treated with surgery or chemoradiotherapy, the survival of patients with advanced cervical cancer is suboptimal, and varying clinical outcomes of cervical cancer patients with the same FIGO stage are also common (Gadducci et al., 2010). Fortunately, reliable biomarkers can be used to assist tumor diagnosis, prognosis, and predict the response to therapy or outcome. Thanks to the progress of bioinformatics techniques, the seeking of promising biomarkers for CESC is more effective. For instance, decreased expression of NUSAP1 and TP73 predicts poor overall survival in cervical cancer (Xie et al., 2020; Ye and Guo, 2019). Additionally, some autophagy-related genes are confirmed as survival prediction signatures in CESC (Chen et al., 2020). Despite the prognosis value of these biomarkers has been revealed, those studies are short of molecular networking analysis, and have no further functional experimental validation, an understanding of the comprehensive molecular mechanisms is lacking. In our study, we found TMEM33 was up-regulated in CESC and most other cancer types. TMEM33 was confirmed as a potential marker for the diagnosis of CESC with high sensitivity and specificity using ROC curve analysis. We determined the predictive value of TMEM33 for OS, PFI, and DSS in CESC patients. Multivariate Cox analysis further confirmed that the high TMEM33 expression was an independent risk factor for OS in patients with CESC. Pathway analysis revealed DEGs were enriched in immune response pathways. Finally, we examined the expression of TMEM33 in cervical cancer cells and uncovered its role in facilitating cell proliferation.

TMEM33 is a conserved protein harboring three transmembrane domains, regulating the tubular ER network (Urade et al., 2014). TMEM33 is highly expressed in breast cancer cells and up-regulated in early recurrent breast cancer specimens compared with non-recurrent breast tumors (Sakabe et al., 2015), however, its expression in the majority of cancers has not been well characterized. Through bioinformatics analysis on TCGA and GTEx datasets, we found TMEM33 was up-regulated in 24 of 33 cancer types compared with normal tissues, indicating TMEM33 might be involved in tumorigenesis. The ROC curve for TMEM33 discrimination of CESC diagnosis had an AUC of 0.881, strongly suggesting that TMEM33 was a potential biomarker for CESC diagnosis. High TMEM33 expression was correlated with height, N stage and histological type. Moreover, cervical cancer patients with higher TMEM33 levels showed strikingly worse OS, PFI, and DSS. The nomogram and calibration curve indicated a possibility that it could represent a prognostic biomarker for cervical cancer.

Given limited previous research with regard to TMEM33 in cancer, we performed functional analysis based on GO, KEGG and GESA in CESC. Interestingly, the DEGs were enriched in immune responses and immunoregulation, in addition to pathways in signal transduction, small molecule biosynthesis, and transmembrane transport. It has been reported that RNF26 co-assembles with TMEM43, TMED1, TMEM33, and ENDOD1 to form a complex, modulating innate immune signaling through the cGAS-STING pathway (Fenech et al., 2020). Here, we revealed the underlying relationship between TMEM33 expression and immune cell infiltration. Tumor-infiltrating immune cells are implicated in tumor progression, metastasis and chemoradiotherapy resistance (Clark et al., 2007). We found TMEM33 expression was positively correlated with T helper cells and eosinophils, but negatively correlated with DCs, Th1 cells, cytotoxic cells, B cells and T cells. As the antigen presenting cells, DCs activate CD8^+^ T cells and then initiate anti-tumor responses (Meyer et al., 2018). Th1 cells play a vital role in anti-cancer immunity. Th1 polarization enhance CTLs-mediated killing of tumor cells and tumor regression (Clever et al., 2016). T cells, especially the CD8^+^ cytotoxic T cells mediated cellular immunity participant in anti-tumor immunity (Xu et al., 2020). Therefore, highly expressed TMEM33 in cervical cancer may potentially impact tumor immunity thereby contributing to oncogenesis.

To further elucidate the underlying biological function of TMEM33, we analyzed TMEM33-interacted and similar genes using STRING and GEPIA2 websites, respectively. The common gene RNF4, is an E3 ubiquitin-protein ligase involved in chromosome alignment, spindle assembly, hypoxia and heat shock responses, and regulation of transcription. Studies found that up-regulated RNF4 stabilizes certain oncoproteins and potentiates tumor cell properties (Thomas et al., 2016). Other five similar genes OCIAD1, TMED5, DHX15, MED28 and LETM1 were also selected according to their role in tumorigenesis, and their correlation with TMEM33 were analyzed. Increased TMED5 expression facilitates cell proliferation, invasion and migration in cervical cancer cells (Yang et al., 2019). LETM1 is a target of miR-613 in suppressing cervical cancer progression (Ji and Hu, 2020). In summary, the molecular networking analysis provided more evidence to deeply understand the potential mechanism of TMEM33 in cervical cancer.

The last issue of interest we addressed was the role of TMEM33 in cervical cancer cells. In zebrafish, TMEM33 mediates the effects of VEGF during angiogenesis and is required for VEGF-mediated cytosolic Ca2+ signaling within endothelial tip cells. TMEM33 knockdown inhibits endothelial cells proliferation in response to VEGF (7). After we examined the expression of TMEM33 in cervical cancer cells, HeLa and SiHa cells were further selected for the TMEM33 functional study. Inhibition of TMEM33 decreased the cell viability, colony formation and DNA synthesis of HeLa and SiHa cells, indicating TMEM33 was involved in cell proliferation regulation. In addition, we examined the mRNA expression of RNF4, OCIAD1, TMED5, DHX15, MED28, and LETM1 in TMEM33 knockdown cervical cancer cells. Five of these genes were significantly decreased in HeLa cells while all six genes were decreased in SiHa cells. Consequently, TMEM33 dysregulation may contribute to the tumorigenesis of cervical cancer.

## Conclusion

Taken together, this is the first study to investigate the oncogenic role of TMEM33 in CESC. High expression of TMEM33 predicted poor prognosis and was considered as an independent prognostic factor and potential therapeutic target for cervical cancer. Increased TMEM33 expression correlated with immune cell infiltration and promoted cell proliferation in cervical cancer. Our findings revealed a novel role of TMEM33 in tumorigenesis of CESC.

## Data Availability

The original contributions presented in the study are included in the article/Supplementary Material further inquiries can be directed to the corresponding authors.

## Footnotes

1 STRING: https://string-db.org/.

2 GEPIA2: http://gepia2.cancer-pku.cn.

3 HPA: https://www.proteinatlas.org
