# Supplementary material for: High Expression of TMEM33 Predicts Poor Prognosis and Promotes Cell Proliferation in Cervical Cancer
Source: Front Genet. 2022 Jun 27;13:908807. doi: 10.3389/fgene.2022.908807 (PMC9271802; doi:10.3389/fgene.2022.908807)
Supplement: Supplementary file 1 [file DataSheet1.docx]

Supplementary Material

## Supplementary Figures


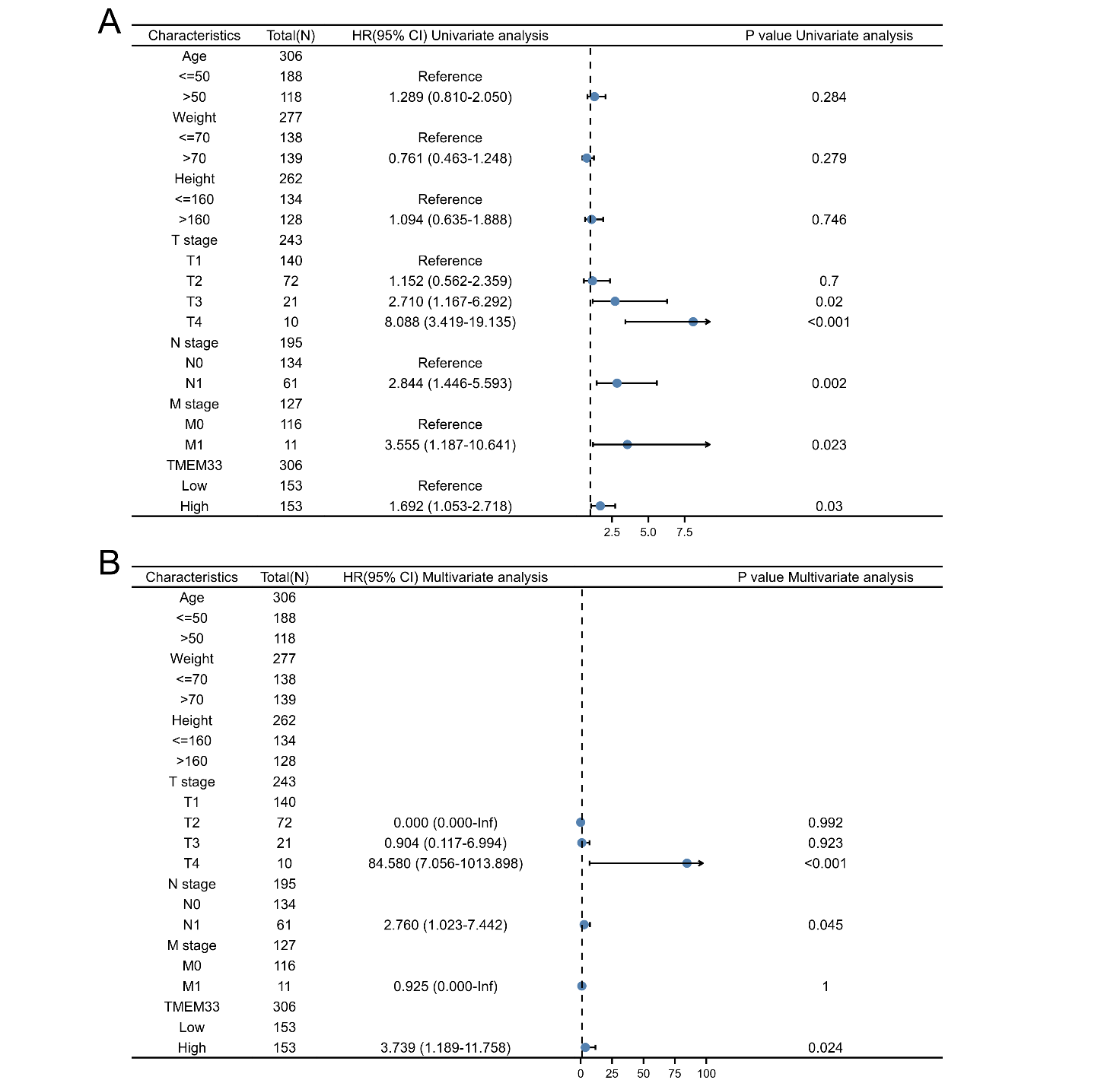


**Supplementary Figure 1.** Univariate and multivariate cox hazard regression analysis. (A) Univariate Cox hazard regression analysis of clinicopathological variables (TMEM33, age, weight, height, T, N, M) in CESC from TCGA dataset. (B) Multivariate Cox hazard regression analysis of clinicopathological variables (TMEM33, age, weight, height, T, N, M) in CESC from TCGA dataset.


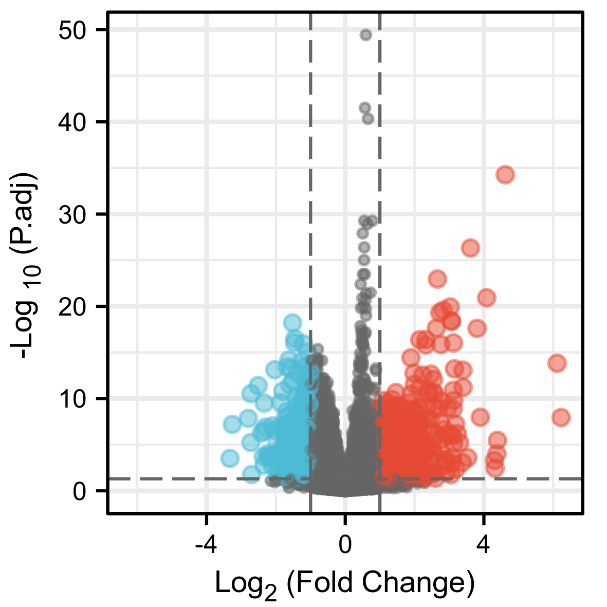


**Supplementary Figure 2.** Volcano plots of DEGs of TMEM33 in CESC obtained from TCGA dataset. The red plots represent positive correlated genes and the blue plots are negative correlated genes (|log2(FC)|>1 & p. adj<0.05).


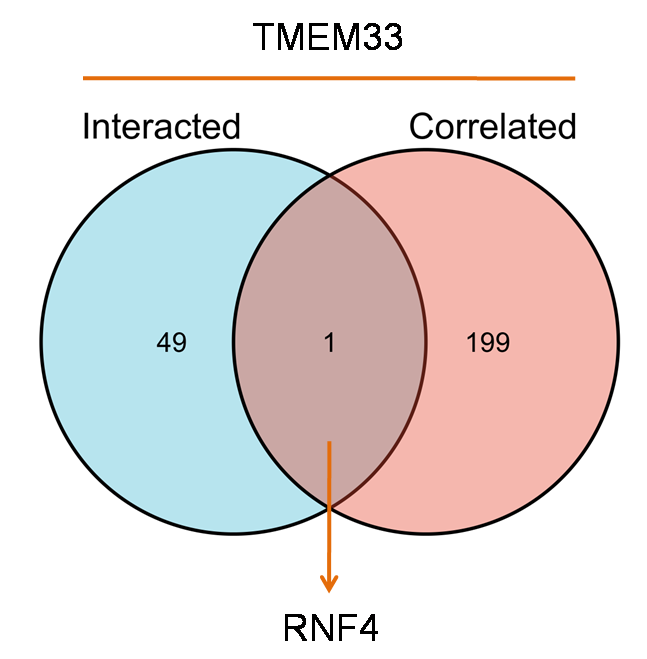


**Supplementary Figure 3.** An intersection analysis of 200 TMEM33-similar genes and 50 TMEM33-interacted genes was shown in Venn diagram.

## Supplementary Tables

**Supplementary table 1**. Oligo sequences used in the present study.

| Method | Oligo name | Sequence (5’-3’) |
| --- | --- | --- |
| RNAi | siTMEM33 | GCUUCAUGCUGCCACAUAUdTdT |
| RNAi | siCon | UUCUCCGAACGUGUCACGUdTdT |
| qPCR | TMEM33 (F) | ACGCAAGGGGCTCAAATAGT |
| qPCR | TMEM33 (R) | TGTCGCAGGCATCAGGAATA |
| qPCR | RNF4 (F) | CTGCATGGACGGATACTCAGA |
| qPCR | RNF4 (R) | TACAGAAGACATGGCCGCATT |
| qPCR | OCIAD1 (F) | AATGGGAGGGCTGATTTTCG |
| qPCR | OCIAD1 (R) | AATCAGGCCCTATGTGGGGA |
| qPCR | TMED5 (F) | GCTTCACACCTTCCCTCGATA |
| qPCR | TMED5 (R) | ACTTGGTACTCGATCTCCAGC |
| qPCR | DHX15 (F) | CCACAAACCTGAATGGGTGC |
| qPCR | DHX15 (R) | GCAATGATGCGGTCCAACTG |
| qPCR | MED28 (F) | TTCGCTTCTTCAGGCAGCTC |
| qPCR | MED28 (R) | ATGACTCCAACTCGTCCACC |
| qPCR | LETM1 (F) | ACTCCCATCCACCCTGTGTA |
| qPCR | LETM1 (R) | CCCACAGAGGTAGAGGTCCA |
| qPCR | GAPDH (F) | GCACCGTCAAGGCTGAGAAC |
| qPCR | GAPDH (R) | TGGTGAAGACGCCAGTGGA |
